# A Reference-Free and Non-Contact Method for Detecting and Imaging Damage in Adhesive-Bonded Structures Using Air-Coupled Ultrasonic Transducers

**DOI:** 10.3390/ma10121402

**Published:** 2017-12-08

**Authors:** Timotius Yonathan Sunarsa, Pouria Aryan, Ikgeun Jeon, Byeongjin Park, Peipei Liu, Hoon Sohn

**Affiliations:** 1 Department of Civil and Environmental Engineering, KAIST, 291 Dahakro, Yuseong-gu, Daejeon 34141, Korea; timotisun@kaist.ac.kr (T.Y.S.); pouria.aryan@kaist.ac.kr (P.A.); j-catcher1230@kaist.ac.kr (I.J.); peipeiliu@kaist.ac.kr (P.L.); 2 Composites Research Division, Korea Institute of Materials Science, 797 Changwondaero, Seongsan-gu, Changwon, Gyeongsangnam-do 51508, Korea; b.park@kims.re.kr

**Keywords:** adhesive bonded structure, air-coupled transducer, debonding, weakened bon, non-destructive testing, reference-free, in situ

## Abstract

Adhesive bonded structures have been widely used in aerospace, automobile, and marine industries. Due to the complex nature of the failure mechanisms of bonded structures, cost-effective and reliable damage detection is crucial for these industries. Most of the common damage detection methods are not adequately sensitive to the presence of weakened bonding. This paper presents an experimental and analytical method for the in-situ detection of damage in adhesive-bonded structures. The method is fully non-contact, using air-coupled ultrasonic transducers (ACT) for ultrasonic wave generation and sensing. The uniqueness of the proposed method relies on accurate detection and localization of weakened bonding in complex adhesive bonded structures. The specimens tested in this study are parts of real-world structures with critical and complex damage types, provided by Hyundai Heavy Industries^®^ and IKTS Fraunhofer^®^. Various transmitter and receiver configurations, including through transmission, pitch-catch scanning, and probe holder angles, were attempted, and the obtained results were analyzed. The method examines the time-of-flight of the ultrasonic waves over a target inspection area, and the spatial variation of the time-of-flight information was examined to visualize and locate damage. The proposed method works without relying on reference data obtained from the pristine condition of the target specimen. Aluminum bonded plates and triplex adhesive layers with debonding and weakened bonding were used to examine the effectiveness of the method.

## 1. Introduction

To fulfill the demand for lighter and more resistant materials in the aerospace, automobile, and marine industries, adhesive bonded structures have been widely adopted [1,2,3]. The use of adhesive bonded structures has become popular due to their ability to provide more uniform load transfer. Improved uniform load transfer in adhesive bonded structures results in better damage tolerance and, more importantly, fatigue life extension, compared to conventional joining methods such as mechanical fastening [4]. 

Problems such as manufacturing flaws or impact damage can, however, adversely affect the mechanical properties of the bonded structures [1]. Inclusions, debonding, and weakened adhesion are three critical damage types that commonly exist in adhesive bonds [4]. Inclusion is defined as the existence of foreign material in the adhesive bond. Debonding is the inclusion of air in adhesive bonds and causes non-uniform loading that can trigger the failure of the structure [4]. Lastly, weakened bonding is caused by impact or improper adhesion between the adhesive and the adhered. Consequently, weakened bonding results in a weaker bond between the adhesives and the adhered, leading to a change in the local mechanical properties of the structure [4]. The presence of weakened bonding is considered a serious problem for bonded structures [5]. The development of non-destructive methods for reliable bonded structure inspection is a subject of intensive research, since non-destructive methods could lead to more efficient and safer structures with lighter weight and lower cost [6].

Several damage detection methods have been developed and considered to address the challenges in the inspection of bonded structures. These methods include neutron imaging radiography [7], thermography [8,9,10], and ultrasound [11,12,13]. Some methods, such as X-ray and thermography, have been confirmed successful for other types of damage but were not sufficiently sensitive to the presence of weakened bonding [6]. 

Ultrasonic methods have been found to be promising for detecting different types of damage and weakened bonding [14,15,16]. However, certain drawbacks limit the possibility of real industrial applications. For example, the ultrasonic method developed in Castaings et al. [12] only works for conductive materials. The ultrasonic method proposed by Cuc and Giurgiutiu [17] requires the attachment of the sensor and actuator on the surface of the structure. The laser “shockwave” technique has been used to detect weakened bonding in composite structures, but the shockwave can damage the structure or the bonding itself [6,18]. Amongst available ultrasonic approaches, non-contact methods using an air-coupled transducer (ACT) [19] and a laser [20] have shown potential for in-situ applications. In a number of studies, ACT systems were applied for damage detection and imaging of a number of structural components made of different materials [19,21,22]. 

This paper presents a fully non-contact, baseline-free candidate method for the in-situ damage detection of adhesive-bonded structures using ACTs. The method uses the time-of-flight (TOF) of the ultrasonic waves over a target inspection area to assess and visualize the presence of critical damage. Aluminum bonded plate and triplex adhesive bond, with debonding and weakened adhesion damage, were used to confirm the effectiveness of the method. The significance of the developed system lies in being a fully non-contact method for various damages and materials, in its damage size estimation and localization, and the potential for in-situ applications.

The remainder of the paper is organized as follows. Section 2 introduces the proposed method including the schematic pictures of the setups and the imaging algorithm. Section 3 discusses the performance of the proposed method for debonding detection of adhesive bonded aluminum plates, and Section 4 considers weakened bonding detection of adhesive bonded triplex layers. Finally, a conclusion is presented in Section 5, summarizing the outcomes and considerations for real industrial applications.

## 2. Method

This section explains the proposed method used to obtain the experimental results for various setup configurations. First, an overview of the method is provided. Next, the detailed description of the imaging algorithm used to visualize the different types of damage is described.

### 2.1. Overview of Air Coupled Ultrasonic Transducer Scanning 

Ultrasonic testing can be performed using two ACTs with several configurations. Through transmission and pitch-catch are the two most common configurations reported in the literature [22,23,24]. 

**Figure 1:**
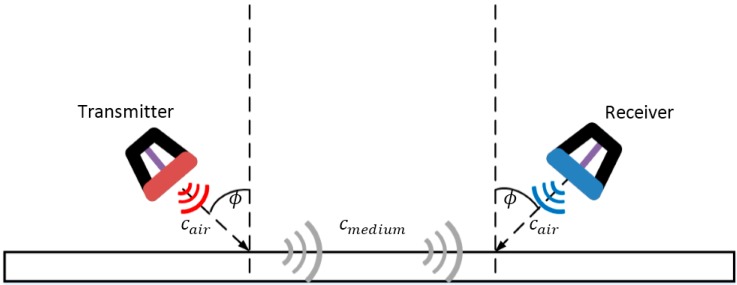
Schematic illustration of the use of Snell’s law to determine the incident and sensing angles of air-coupled transducers (ACTs) in the pitch-catch configuration.

The ultrasonic waves generated in a pitch-catch configuration are in the form of guided waves. The guided waves travelling in thin plates are dispersive and multimodal [23,24]. Hence, to excite a desired wave mode, the incident angle of the ACT has to be set to a specific value based on Snell’s law [22]. Figure 1 illustrates how to use Snell’s law to determine the incident and sensing angles of ACTs in the pitch-catch configuration.

Snell’s law describes the relationship between the angles of incidence and sensing when the ultrasonic waves pass through a boundary between two different media. This concept is explained in Equation (1).

(1) $$\frac{\sin\phi }{\sin\theta }=\frac{C_{air}}{C_{medium}}$$

where ϕ and θ denote the angles of incidence and refraction, respectively, and $C_{air}$ and $C_{media}$ represent the velocities of the ultrasonic waves in air and in the target medium, respectively. As the ultrasonic waves propagate along the surface θ=90° (sin90°=1), the incident angle ϕ can be calculated. As for sensing, the sensing angle can be set equal to the incident angle to measure the identical wave mode.

Since different guided wave modes travel at different wave velocities, choosing the appropriate incident and sensing angles allows the selective measurement of a desired mode of the guided waves. If the thickness and material properties of the plate are known, the incident and sensing angles can be easily calculated using theoretical dispersion curves. Otherwise, the velocity of the mode of interest can be measured experimentally.

### 2.2. Wavelet Transform for Extracting Time of Flight 

Wavelet transform was used to estimate the time of flight (TOF) of the measured signal [25]. The wavelet transform maps a time function g(t) into a two-dimensional function $\psi_{s,\tau }\left(t\right)$:

(2) $$\psi_{s,\tau }\left(t\right)=\frac{1}{\sqrt{s}}\psi \left(\frac{t-\tau }{s}\right)$$

where $\psi_{s,\tau }\left(t\right)$ is a family of wavelets created from a mother wavelet ψ(t). The complex Morlet wavelet, obtained as the product of a complex exponential and a Gaussian window, was used as the mother wavelet ψ(t). s and τ denote the scale and translation parameters, respectively. s scales ψ(t) by compressing or stretching it, and τ translates ψ(t) along the time axis. For a given time function g(t), the continuous wavelet transform (CWT) at scale s and position τ is the convolution of the time function g(t) with the wavelet function $\psi_{s,\tau }\left(t\right)$:

(3) $$CWT\left(s,\tau \right)=\int_{-\infty }^{+\infty }g\left(t\right)\cdot \psi_{s,\tau }\left(t\right)dt$$

where CWT(s,τ) is a wavelet coefficient of g(t) for the given s and τ values. The wavelet coefficient represents the energy of *g*(*t*) at the given s and τ values. The time position and the scale for the maximum amplitude of g(t) can be obtained through the aforementioned wavelet analysis. Therefore, the TOF and the group velocity of the measured guided wave were estimated from the wavelet analysis of the acquired signal. The TOF was extracted at different input frequencies (*f*), and the input frequency range was defined based on the frequency response of the test specimen. The following equation provides the definition of the proposed feature:

(4) $$\mathrm{Feature}=\underset{f}{\sum }TOF_{f}$$

### 2.3. Imaging Algorithm

**Figure 2:**
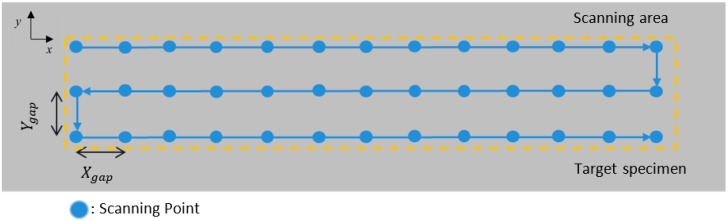
Illustration of the synchronized scanning for both through transmission and pitch-catch configurations.

A synchronized scanning strategy was developed to localize damage on the inspected target specimens. Figure 2 illustrates the scanning process used for both through transmission and pitch-catch configurations. An inspection point in the through transmission configuration is the point where the propagating waves intersect with the specimen, whereas in the pitch-catch configuration, the inspection point is the midpoint along the wave propagation path between the transmitter and the receiver. The inspection point is shifted along the scanning area according to the scanning sequences. “Scan gap” is defined as the distance between two adjacent inspection points, and this gap constitutes the inspection resolution within the scanning area. *X*_gap_ and *Y*_gap_ denote the scan gaps in the *x* and *y* directions, respectively. Based on the target specimen and the damage type, the distance between the transmitter, the receiver, and the scan gap can be adjusted to optimize the scanning procedure.

To detect damage using the synchronized scanning strategy, the proposed feature needs to be extracted from the acquired signals. Depending on the type of the target specimen and damage, different features, such as signal amplitude, phase, velocity, and mode conversion of ultrasonic waves, can be extracted [26].

**Figure 3:**
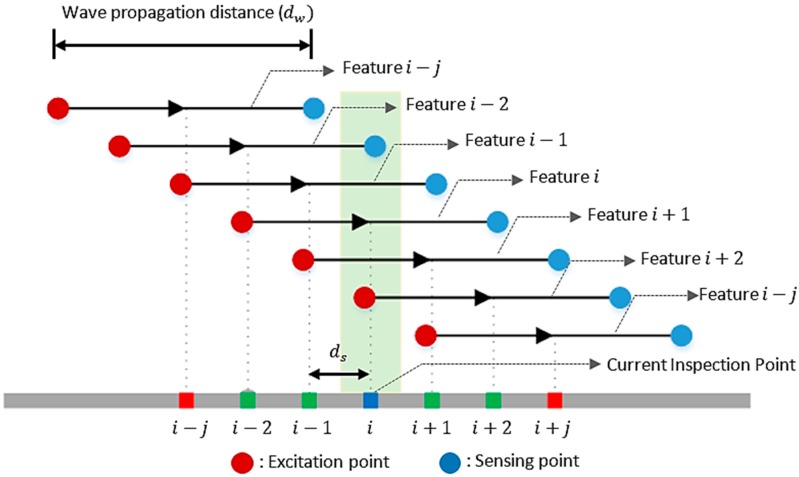
Calculation of damage index (DI) for the *i*th inspection point in the scanning area [25].

The summation of the TOF extracted at different frequencies is defined as the proposed feature, as presented in Equation (4). By calculating a damage index (DI) for the ultrasonic signal, measured from each inspection point, damage within the target specimen can be visualized. At a specific inspection point, the DI is obtained by performing a weighted summation of the features from near-by inspection points as follows:

(5) $$DI_{i}=\underset{j}{\sum }W_{i\pm j}\times feature_{i\pm j},j=0,1,2,3,\ldots$$

where $feature_{i\pm j}$ denotes the feature value extracted from Equation (4) and corresponds to the *i*th inspection point or its spatially adjacent *i ± j*th points, as shown in Figure 3. Since the wave propagation path of one inspection point can overlap the wave propagation path of another inspection point, a weight value, $W_{i\pm j}$, for each inspection point was defined based on the overlapping condition between the wave propagation distances related to the adjacent points as:

(6) $$W_{i\pm j}=\{\begin{array}{cc} {\left(1-\frac{j\times d_{s}}{0.5\times d_{w}}\right)}^{2} & if\;\left(j\times d_{s}\right)<\left(0.5\times d_{w}\right)\\ 0 & if\;\left(j\times d_{s}\right)\geq \left(0.5\times d_{w}\right) \end{array}$$

where $d_{w}$ and $d_{s}$ are the distances of the wave propagation and the scan gap, respectively. Equation (6) describes the conditions where the overlapping occurs. Equation (6) states that the overlapping depends on the distances of the wave propagation, which is the distance between excitation and sensing points, and the scan gap. Figure 3 illustrates the calculation of the damage index for each inspection point in the scanning area. By examining the spatial distribution of the DI values over the entire scanning area, the damage area with abnormal DI values can be detected and localized. Because of the spatial comparison of DI values, the proposed imaging technique does not rely on any baseline data obtained from the pristine condition of the target specimen.

## 3. Adhesive Bonded Aluminum Plates with Debonding

This section presents the performance of the proposed method to detect debonding in adhesive bonded aluminum plates. First, the target specimens and the experimental setup are described. Next, the obtained results are presented.

### 3.1. Specimen Description

**Figure 4:**
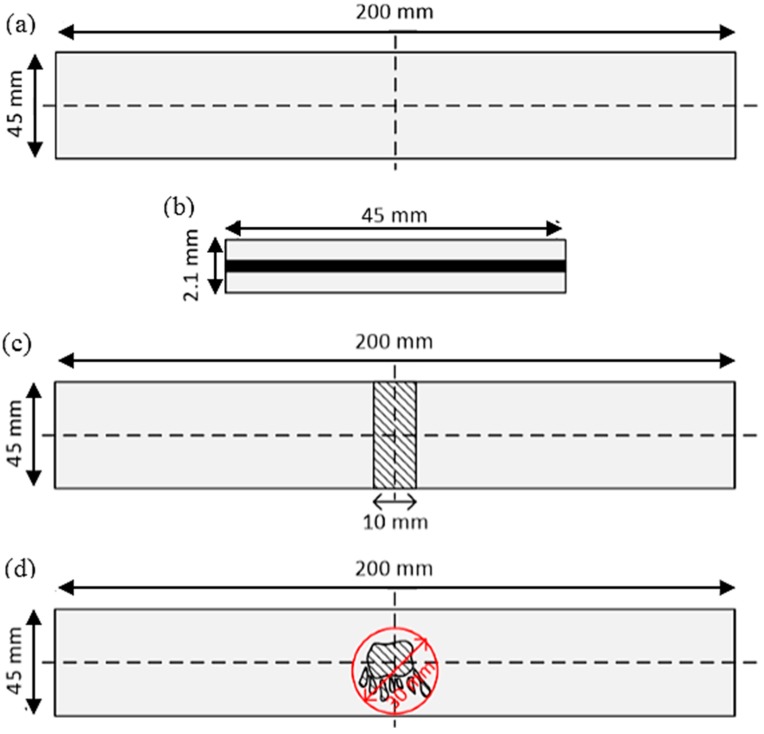
Specimen geometrical information: (**a**) top view of the adhesive bond aluminum plate; (**b**) cross-sectional view of the target specimen; (**c**) debonding area of adhesive bonded aluminum plate 1; and (**d**) debonding area of adhesive bonded aluminum plate 2.

Two adhesive bonded aluminum (Al) plates, with a length of 200 mm, a width of 20 mm, and a thickness of 2.1 mm, were bonded together using an adhesive layer. The aluminum plate was made of Al alloy (AlMg^3^ grade), and Betamate was used as an adhesive to join the two aluminum plates. Figure 4 explains the detailed geometrical information of the target specimens. Adhesive bonded aluminum plate 1 had line debonding damage in the middle of the specimen, whereas adhesive bonded aluminum plate 2 had random-shaped debonding damage in the middle of the specimen. IKTS Fraunhofe^®^ provided the specimens.

**Figure 5:**
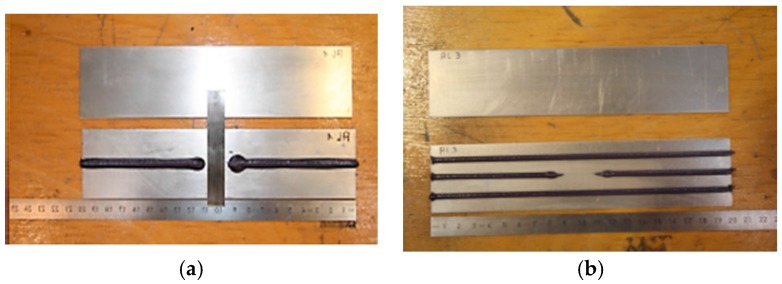
The application of adhesive to the aluminum plates while creating debonding damage: (**a**) adhesive bonded aluminum plate 1; and (**b**) adhesive bonded aluminum plate 2.

The debonding was introduced in the adhesive bond of two aluminum plates by not applying the adhesive before the two aluminum plates were joined. Figure 5 shows the method by which the debonding damages were introduced for each target specimen.

### 3.2. Description of Test Setup

**Figure 6:**
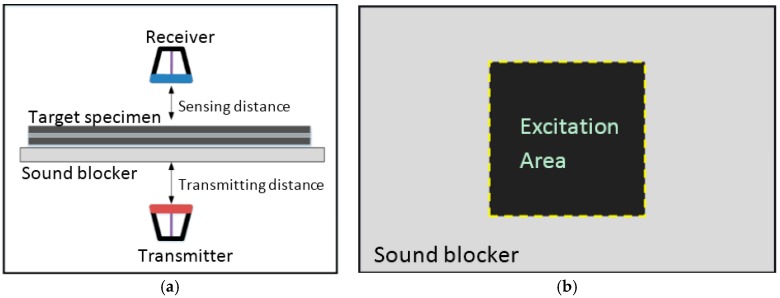
Details of the schematic experimental configuration for the adhesive bonded aluminum plate: (**a**) through transmission configuration for debonding detection; and (**b**) bottom view of the sound blocker. The excitation area denotes a hole for transmitting the ultrasonic wave to the target specimen.

Two ACTs were used to inspect the adhesive bonded aluminum plates. The transmitter (NCG200-D25-P76, the Ultran Group, New York, NY, USA) and the receiver (MicroAcoustic BAT-1, MicroAcoustic Instruments Inc., Quebec, QC, Canada) were set based on the through transmission configuration. The transmitter has a focused circular active transmitting area with a diameter of 25 mm and a central frequency of 200 kHz. The receiver has a 10 mm circular active sensing area, and can measure ultrasonic waves within a frequency band of 40 kHz to 2.25 MHz. The distances from the transmitter and receiver to the specimen surface were fixed at 60 mm and 30 mm, respectively. The distances were fixed in a line, perpendicular to the target specimen. Figure 6a illustrates the details of the experimental configuration. A sound blocker, as described in Figure 6b, was used to prevent leaking waves and also functioned as a support for the specimen with a rectangular opening for transmitting the ultrasonic waves. In the experiment, the receiver and the transmitter were fixed to a firm stand and the specimen was moved.

In this experiment, a 70 V, 5-cycle toneburst signal with a central frequency of 200 kHz was transmitted from the Arbitrary Waveform Generator (AWG) (Agilent Technologies, Santa Clara, CA, USA) to the transmitter. The source signal was generated by using a 33220A Agilent AWG and amplified up to 100 Vpp using an EPA-104 PIEZO Linear Amplifier (PIEZO SYSTEMS, Woburn, MA, USA). The generated signal was monitored by a LeCroy waveRunner 44Xi 400 MHz oscilloscope (Teledyne LeCroy, New York, NY, USA). The signal received by the receiver was amplified by a Q-Amp transimpedance preamplifier (MicroAcoustic Instruments Inc., Ottawa, ON, Canada). All the parts of the equipment were synchronized by a control system consisting of a personal computer and a junction box of PSV-400-3D Polytech. 

**Figure 7:**
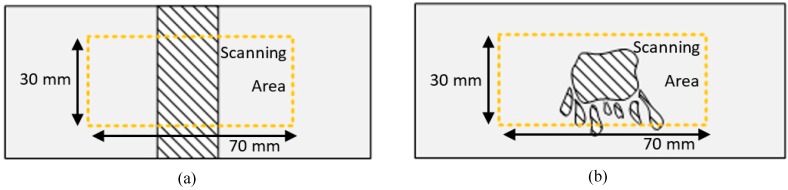
Schematic illustration of the scanning area of adhesive bonded aluminum (**a**) plate and (**b**) plate 2.

The scanning area was set to be a 30 mm × 70 mm rectangular area, as shown in Figure 7. A total of 21 (3 × 7) inspections were assigned with a scan gap, from center to center, of 10 mm in the *y* direction and 20 mm in the *x* direction within the scanning area. To improve the signal to noise ratio, the responses from each inspection point were measured 200 times and averaged in the time domain.

### 3.3. Test Results

**Figure 8:**
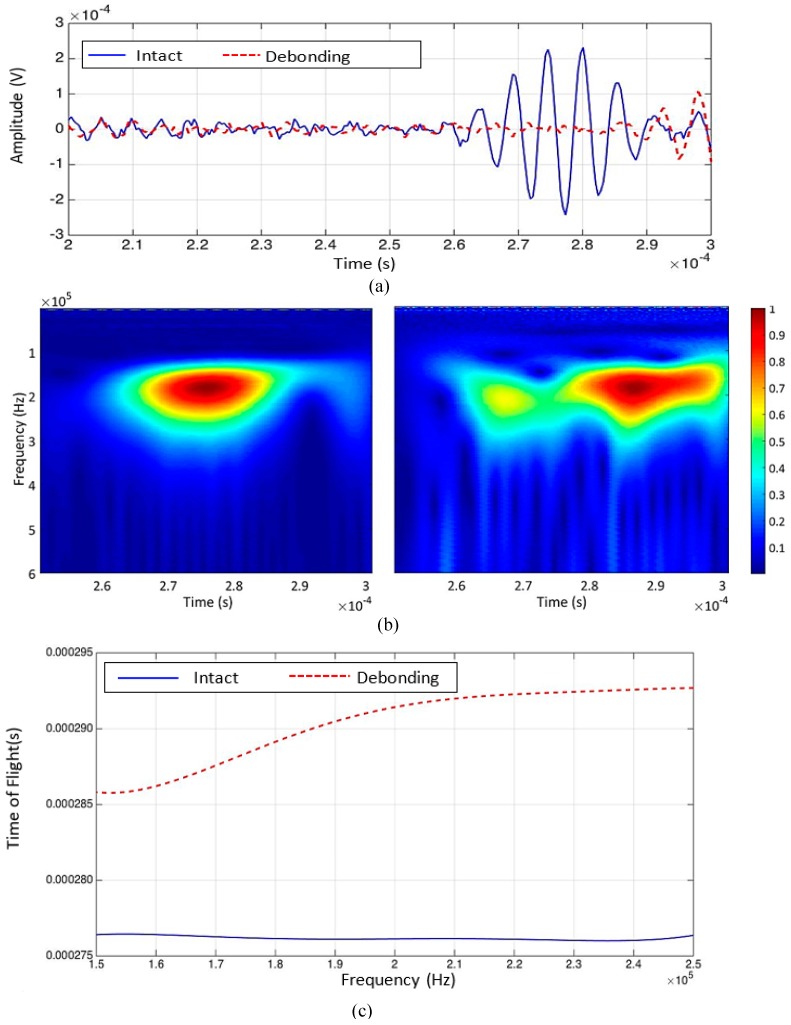
Experimental results: (**a**) two representative signals obtained from intact bonding (blue solid line) and weakened bonding (red dashed line) conditions in the time domain; (**b**) two representative signals obtained from intact (left) and debonding (right) conditions in the time-frequency domain; (**c**) extraction of time of flights (TOFs) from intact (blue solid line) and debonding (red dashed line) conditions within the frequency range of interest.

Two representative signals obtained from the intact and debonding area are displayed in Figure 8a. The corresponding wavelet transform results are shown in Figure 8b. The TOF of the measured wave packet at each frequency was extracted in the time-frequency domain. In Figure 8c, the TOF of the measured signal was extracted in the excitation frequency range of 150 to 200 kHz, and the variation of the TOF due to the presence of debonding damage is presented. The extracted TOF shows that the ultrasonic wave traveling through the debonding damage was traveling slower than the ultrasonic wave traveling through the intact area. The bulk longitudinal wave travels in the air with a velocity equal to 340 m/s, while the bulk longitudinal wave traveled in the adhesive material with a velocity of 2610 m/s. Since debonding is the inclusion of air in the adhesive as described in Section 1, the wave travels faster in the intact region. 

**Figure 9:**
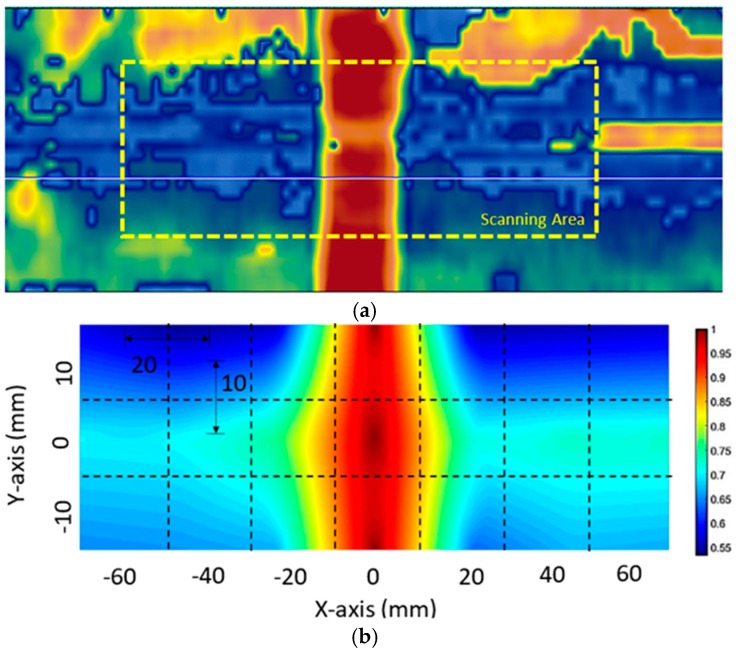
Visualization of the adhesive bonded aluminum plate 1: (**a**) C-Scan result and (**b**) results obtained using the proposed imaging algorithm.

**Figure 10:**
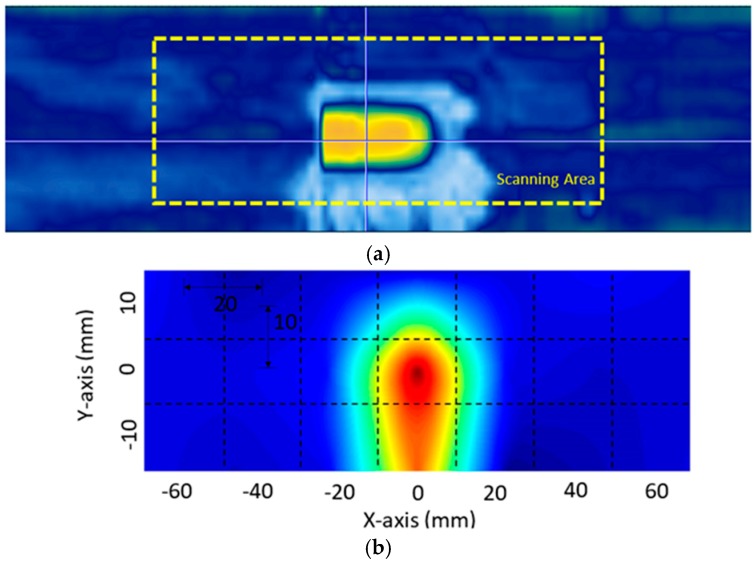
Visualization of the adhesive bonded aluminum plate 2: (**a**) C-Scan result and (**b**) result obtained using the proposed imaging algorithm.

Based on the imaging algorithm described in Section 2, Figure 9 and Figure 10 show the visualization results obtained from the two adhesive bonded aluminum plates. Here, for all the inspection points within the scanning area of each specimen, the corresponding DI values were normalized with respect to the highest DI value within the scanning area. For comparison, a C-Scan image is also provided along with the proposed visualization result. In each C-scan image, the scanning area corresponding to the proposed scanning system is marked with a yellow dashed box.

In the debonding cases, the C-Scan images were used for validation purposes. Compared to the C-Scan images, similar damage patterns and locations of the debonding were observed for both bonded aluminum plates. A distinct difference between the C-Scan image and the obtained results is that the obtained results offer clearer and more promising images for determining the location of the debonding. In addition, the quality of the damage visualization was confirmed to be reliable and the proposed method provides good and accurate insight into the debonding location.

## 4. Adhesive Bonded Triplex Layers with Weakened Bonding

In this section, the performance of the proposed method in weakened bonding detection for adhesive bonded triplex layers is presented. Initially, the target specimen and the setup are described. Then, the results are presented and a discussion of the obtained results is provided. 

### 4.1. Specimen Description

**Figure 11:**
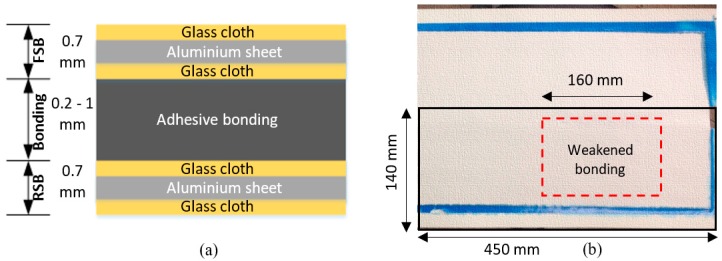
The schematic illustration of the adhesive bonded triplex layers: (**a**) the thickness layout of the adhesive bonded triplex layer; and (**b**) top view of the triplex specimen.

Adhesive bonded triplex layers are part of a secondary barrier in a liquefied natural gas (LNG) carrier cargo tank. The adhesive bonded triplex layers consist of three sub-layers: a flexible secondary barrier (FSB), an epoxy/urethane-based bonding layer, and a resin-based rigid secondary barrier (RSB). Figure 11a shows the adhesive bonded triplex layers. 

Both FSB and RSB sub-layers were composed of a 0.7-mm thick sheet of aluminum with a layer of glass cloth on either side, attached using a resin system. This resin system later determines the rigid nature of the triplex material [4]. The weakened bonding was created by introducing a very thin vinyl between the adhesive and adhered interface. The adhesive bonded triplex layers used in this experiment are shown in Figure 11b. The specimen was provided by Hyundai Heavy Industries^®^, Ulsan, Korea.

### 4.2. Description of Test Setup

Tests were performed using the pitch-catch configuration. No effective non-destructive method currently exists that can detect weakened bonding damage within this type of specimen, as discussed in Section 1. The pitch-catch configuration was chosen for this case, as only the top surface of the triplex layers was accessible for in-situ inspection.

**Figure 12:**
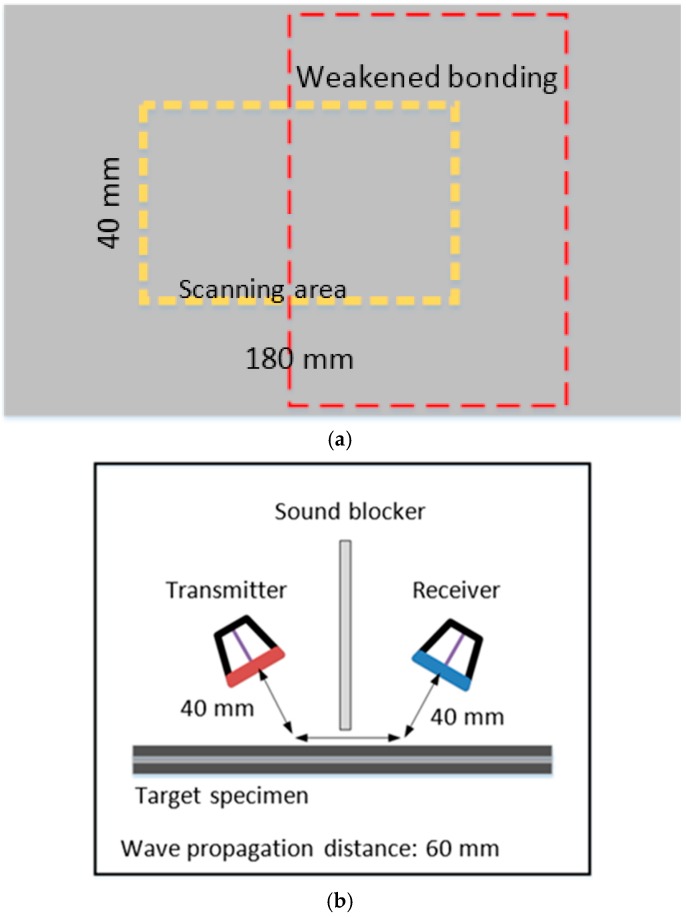
(**a**) Schematic view of the dimensions of the scanning area and location of weakened bonding damage; (**b**) A schematic illustration of the pitch-catch configuration area for weakened bonding detection in the adhesive bonded triplex.

In the experiment, a 100 V, 5-cycle toneburst signal, with a center frequency of 200 kHz was transmitted from the AWG to the transmitter. The ACTs used as the transmitter and receiver were identical to the previous experiment. The schematic diagram of the experiment setup is shown in Figure 12b. The distance between the transmitter and the receiver was fixed at 60 mm, and the lift-off distance for both transducers was set at 40 mm. A sound blocker, made from a triplex board paper, was placed between the transmitter and the receiver to prevent direct reflection and leaking waves from the transmitter. The scanning area was set to be a 40 mm by 180 mm rectangular area, as shown in Figure 12a. A total of 40 (4 × 10) inspection points were defined within the scanning area. The scan gap was set to be 10 mm in the *y* direction (*Y*_gap_) and 20 mm in the *x* direction (*X*_gap_).

To improve the signal to noise ratio, the responses from the transmitting waves were measured 1000 times and averaged in the time domain during scanning. To determine the incident and sensing angles of the ACTs, an experiment was performed on the target specimen using piezoelectric transducers (PZT). The wave velocity of the transmitted wave was calculated by dividing the distance between the transducer and receiver by the TOF. Once the wave speed was estimated, the incident and sensing angles were calculated based on Snell’s law, as shown in Equation (1). A 20 V, 5-cycle toneburst signal, with a central frequency ranging from 155 to 255 kHz with a 10 kHz interval, was transmitted. 

**Figure 13:**
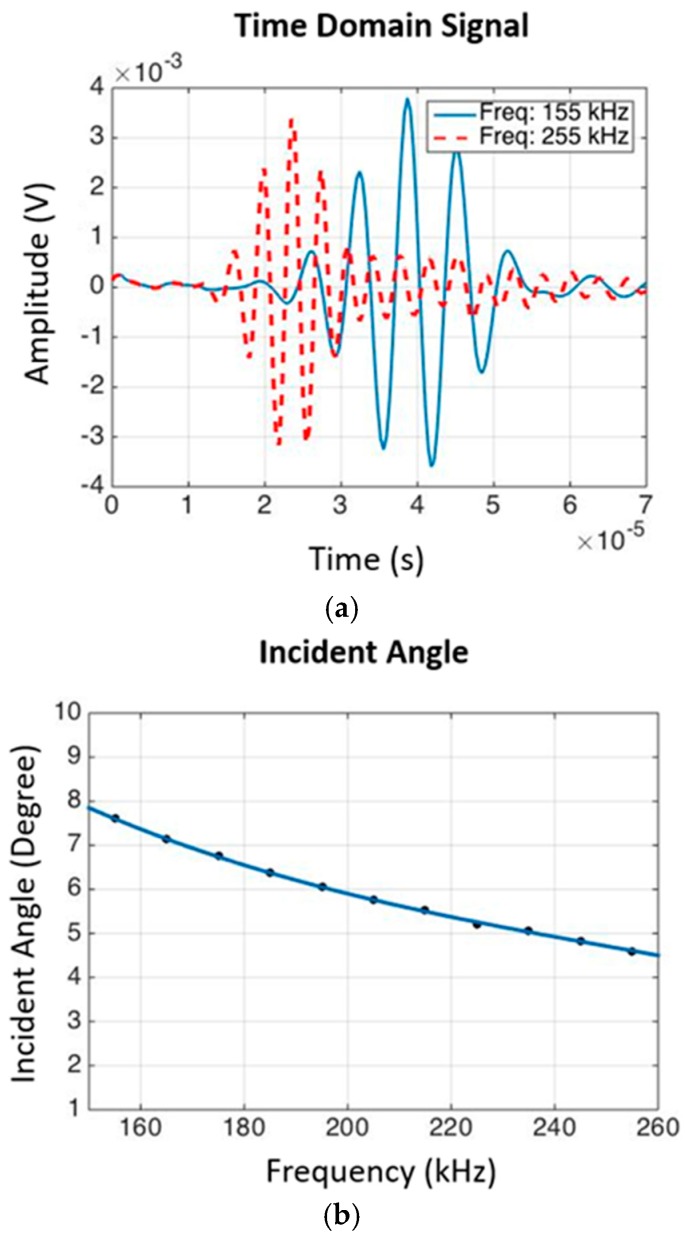
(**a**) Time domain signals of the transmitted anti-symmetric wave. The wave mode with a higher frequency travels with a higher speed; (**b**) Incident angle within the frequency range of interest.

The results of the experiment to determine the incident and sensing angles of the ACTs are presented in Figure 13. According to the calculation in Equation (1) and based on the TOF of the wave packets, the incident and sensing angles of the ACTs should be within the range of 5° to 8°. In this experiment, the incident and sensing angles were set to 5°.

### 4.3. Test Results

**Figure 14:**
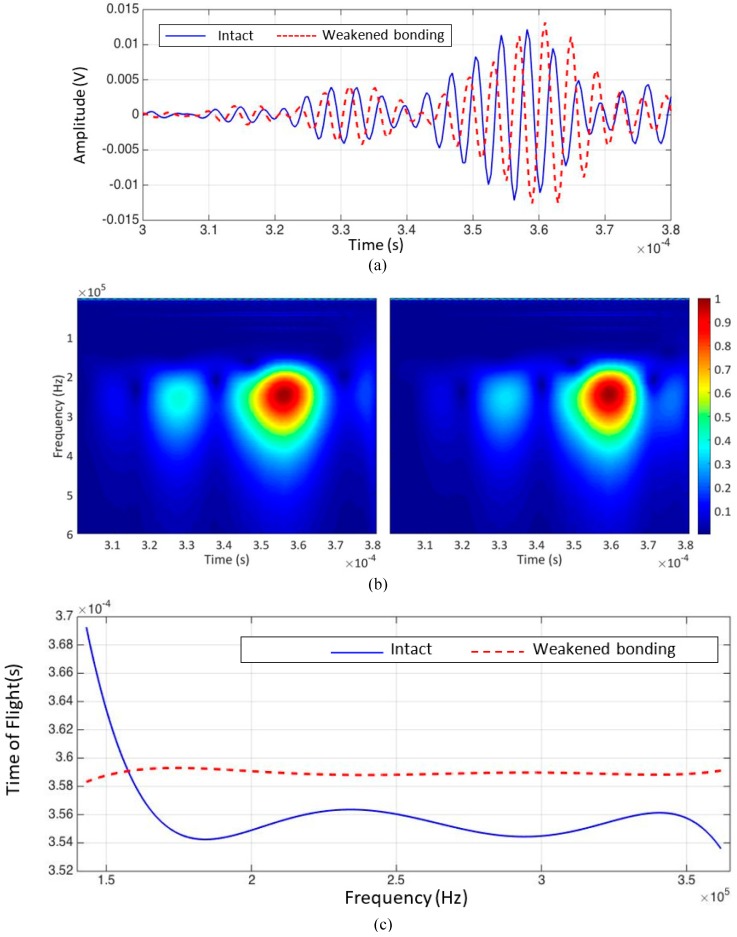
Experimental results: (**a**) two representative signals obtained from intact bonding (blue solid line) and weakened bonding (red dashed line) conditions in the time domain; (**b**) two representative signals obtained from intact (left) and weakened bonding (right) conditions in the time-frequency domain, and (**c**) extraction of TOFs from intact (blue solid line) and weakened bonding (red dashed line) conditions within the frequency range of interest.

Two representative signals obtained from the intact and weakened bonding areas are displayed in Figure 14a. The CWT results are shown in Figure 14b, and the TOF of the measured wave at each frequency is shown in Figure 14c. 

**Figure 15:**
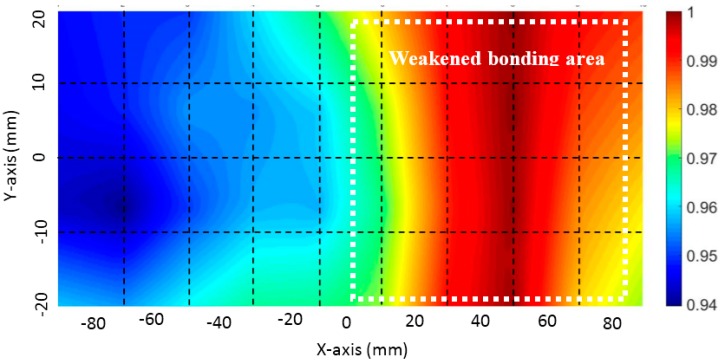
Imaging result of the adhesive bonded triplex layer using continuous wavelet transform (CWT). The white rectangular box shows the actual location of the weakened bonding provided by Hyundai Heavy Industries^®^.

In Figure 14c, the TOF variation in the response signals obtained from the intact and weakened bonding areas are presented as a function of the frequency. The overall velocity of the guided waves measured from the weakened bonding area was significantly lower than the velocity of the guided waves measured from the intact area. The presence of several guided wave modes can be seen in Figure 14b and the TOF from each inspected point was extracted from the wave mode with the highest energy. Figure 15 presents the visualization results obtained using CWT. The actual location of the weakened bonding is marked with the white dashed line provided from the manufacturer Hyundai Heavy Industries^®^.

As with the previous test results in Section 3.3, the high DI values obtained from the pitch-catch configuration signify the location of the weakened bonding. Imaging results obtained by CWT are promising for the clear detection and localization of the weakened bonding. With both configurations, the proposed method not only successfully detected the damage but also visualized the location of the damage. The results from Section 3.3 and Section 4.3 show the effectiveness of the presented method to detect and localize various critical structural damages for adhesively bonded structural components commonly used in aerospace, automobile, and marine industries.

## 5. Conclusions

In this study, a fully non-contact experimental and analytical damage detection candidate method for the in-situ inspection and damage detection of adhesive-bonded structures was developed using air-coupled ultrasonic transducers (ACTs). A synchronized scanning strategy for damage visualization was developed for both through transmission and pitch-catch configurations. The time-of-flight (TOF) information extracted using CWT analysis was defined as a damage sensitive feature for both debonding and weakened bonding detection. The extracted TOF showed that the ultrasonic waves traveling through the debonding damage and weakened bonding damage travel slower than the ultrasonic wave traveling through the intact area. The results confirm that the developed method is successful in detection and localization of debonding damage in adhesive bonded aluminum plates and weakened bonding in adhesive bonded triplex layers. Many studies have indicated that the industries are demanding more reliable non-destructive methods to inspect adhesive bonded structures to ensure the quality of the structure [7,8,11,26]. The specimens tested in this study were parts of real-world structures, provided by Hyundai Heavy Industries^®^, Ulsan, South Korea and IKTS Fraunhofer^®^, Hermsdorf, Germany, with critical and complex damage types. Testing of real-world structural components adds to the significance of the current study. Reliable practical inspection methods can result in huge cost reductions and improved safety of the structures. This study was partially motivated by these expectations and presents a candidate method for the in-situ inspection and damage detection of adhesive-bonded structures.

In particular, the proposed method is a suitable candidate for in-situ applications because it can be flexibly operated with various configurations, and the method does not rely on reference data to assess the presence of critical damage. In other words, the proposed method is independent of the variation of the environmental and operational conditions. A further study is warranted to improve the scan speed and post processing as well as the scan time for field development.
